# Successful Conversion Surgery for Locally Advanced Pancreatic Neuroendocrine Carcinoma: A Case Report

**DOI:** 10.70352/scrj.cr.24-0064

**Published:** 2025-03-20

**Authors:** Yuuko Tohmatsu, Mihoko Yamada, Nobuyuki Ohike, Tomoko Norose, Hidemasa Kubo, Ryo Ashida, Katsuhisa Ohgi, Shimpei Otsuka, Yoshiyasu Kato, Hideyuki Dei, Katsuhiko Uesaka, Takashi Sugino, Teiichi Sugiura

**Affiliations:** 1 Division of Hepato-Biliary-Pancreatic Surgery, Shizuoka Cancer Center, Sunto-gun, Shizuoka, Japan; 2 Division of Pathology, Shizuoka Cancer Center, Sunto-gun, Shizuoka, Japan; 3 Department of Pathology, St. Marianna University School of Medicine, Kawasaki, Kanagawa, Japan

**Keywords:** pancreatic neuroendocrine carcinoma, conversion surgery, *KRAS* mutation

## Abstract

**INTRODUCTION:**

Pancreatic neuroendocrine carcinoma (panNEC) is a poorly differentiated, highly malignant neoplasm with an extremely poor prognosis. This tumor often presents as locally advanced or unresectable at the initial diagnosis.

**CASE PRESENTATION:**

A 72-year-old woman presented to our hospital with weight loss. A computed tomography scan showed an enhanced tumor measuring 32 mm in the pancreatic head region, with contact to the common hepatic artery over 180°. The pathological findings from the specimens obtained via endoscopic ultrasonography-guided fine-needle aspiration identified small cell-type NEC with extensive necrosis, leading to a diagnosis of locally advanced unresectable panNEC. Accordingly, she began a course of carboplatin and etoposide therapy. After 7 courses, given the significant shrinkage of the tumor, we performed a pancreatoduodenectomy as a conversion surgery. Pathological examination revealed a localized, residual nodule of NEC, consisting mainly of large neoplastic cells, with carcinoma *in situ* components scattered within and around the nodule. In addition, the diffuse membrane expression of somatostatin receptor 2 was observed in NEC components. Each component showed the same type of *KRAS* mutation (p.G12V) and was considered to originate from a single primary tumor in the pancreas. She received 3 courses of the same regimen as adjuvant chemotherapy and has remained recurrence-free for 24 months.

**CONCLUSION:**

This is a rare case of successful conversion surgery for locally advanced, unresectable panNEC after chemotherapy, providing several important histopathological and molecular insights.

## Abbreviations

CHA
: common hepatic artery
CIS
: carcinoma in situ
CT
: computed tomography
EUS-FNA
: endoscopic ultrasonography-guided fine-needle aspiration
FDG-PET/CT
: ^18^F-fluorodeoxyglucose positron-emission tomography/CT
H&E
: hematoxylin–eosin
NSE
: neuron-specific enolase
PanINs
: pancreatic intraepithelial neoplasias
panNEC
: pancreatic neuroendocrine carcinoma
PDAC
: pancreatic ductal adenocarcinoma
SpA
: splenic artery
SSTR2
: somatostatin receptor 2
UICC
: Union for International Cancer Control

## INTRODUCTION

Pancreatic neuroendocrine neoplasms are epithelial tumors with neuroendocrine differentiation, classified into well-differentiated (pancreatic neuroendocrine tumor [panNET] G1, G2, and G3) and poorly differentiated (pancreatic neuroendocrine carcinoma [panNEC]). Both panNET G3 and panNEC are aggressive (Ki-67 index of over 20%) but differ in their differentiation type, genetic variants, and prognosis.^1)^ At the time of initial diagnosis, panNEC is often locally advanced and unresectable. Even when curative resection is achieved, the prognosis after surgery is extremely poor.^2,3)^ The indications for surgery for panNEC are not well established. While some reports suggest that preoperative chemotherapy followed by definitive surgery could be beneficial in select cases,^4,5)^ the use of preoperative therapy for panNEC is still limited.

In this case report, we describe conversion surgery for locally advanced, initially unresectable panNEC that responded favorably to chemotherapy. Furthermore, histopathological and molecular findings from this case provided some insights into the development of panNEC and its response to treatment.

## CASE PRESENTATION

**Fig. 1:**
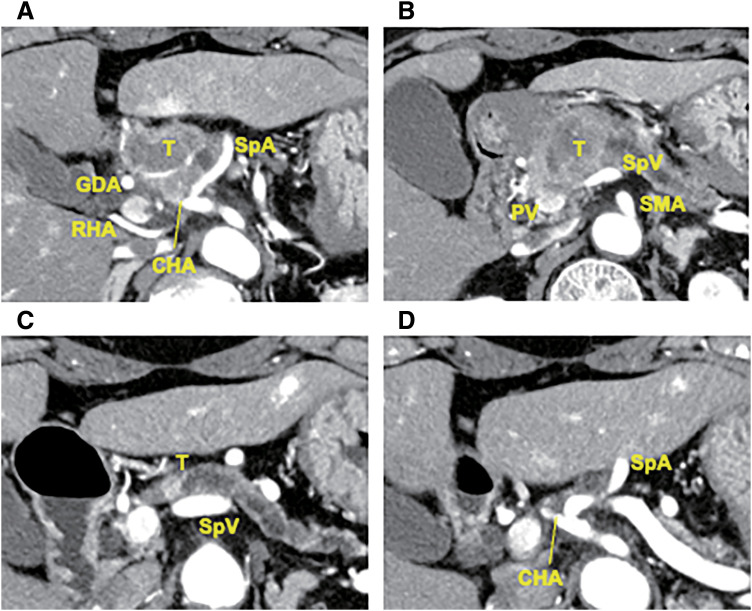
Contrast-enhanced abdominal CT scan before (**A**, **B**) and after neoadjuvant chemotherapy (**C**, **D**). (**A**) A heterogeneously enhanced tumor measuring 32 mm was observed in the pancreatic head region, with possible involvement of the common hepatic artery over 180°. (**B**) The root of the splenic vein was invaded. (**C**) The tumor size was significantly reduced to 8 mm. (**D**) The tumor was no longer in contact with the arteries. CHA, common hepatic artery; CT, computed tomography; GDA, gastroduodenal artery; PV, portal vein; RHA, right hepatic artery; SMA, superior mesenteric artery; SpA, splenic artery; SpV, splenic vein; T, tumor

**Fig. 2:**
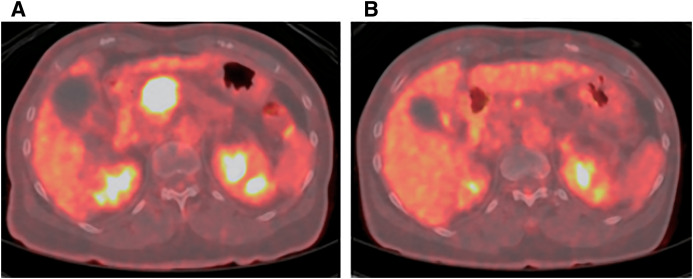
FDG PET/CT before (**A**) and after (**B**) chemotherapy. (**A**) FDG uptake in the pancreatic body tumor increased, with a maximum standard uptake value of 11.6. (**B**) After chemotherapy, the FDG uptake decreased, with a maximum standard uptake value of 3.0. FDG, ^18^F-fluorodeoxyglucose; PET/CT, positron-emission tomography/computed tomography

A 72-year-old woman was referred to our hospital with a history of weight loss. She had a history of diabetes, with an elevated hemoglobin A1c level of 7.7%. Her neuron-specific enolase (NSE) level was markedly elevated at 45.9 ng/mL, while CA19-9, DUPAN-2, and Span-1 were within normal limits. An enhanced abdominal computed tomography (CT) scan showed a heterogeneously enhanced tumor measuring 32 mm in the pancreatic head region (**Fig. 1A**). The tumor exhibited over 180° of contact with the common hepatic artery (CHA) and had also contacted the celiac artery, splenic artery (SpA), left gastric artery, and right hepatic artery. The root of the splenic vein was invaded as well (**Fig. 1B**). No distant metastases were found in other organs. ^18^F-fluorodeoxyglucose (FDG) positron-emission tomography/CT (PET/CT) showed increased FDG uptake in the pancreatic head tumor, with a maximum standard uptake value of 11.6 (**Fig. 2A**).

**Fig. 3:**
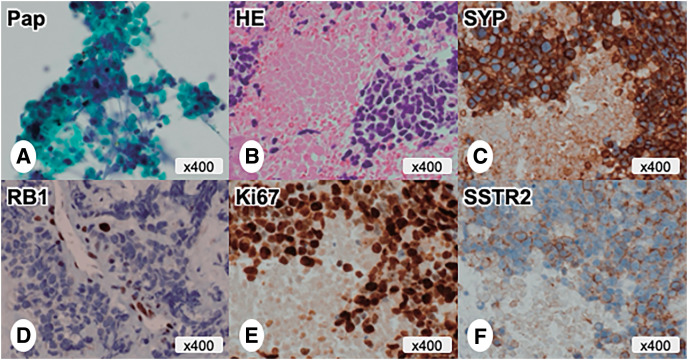
Pathological and immunohistochemical findings of EUS-FNA and EUS-FNB before chemotherapy. (**A**) Cytological specimens with Pap stain showed several cell clusters consisting of small round atypical cells and necrotic cells. (**B**) Histological specimens with H&E stain showed numerous hypercellular clusters composed of small, round atypical cells and extensive necrotic areas. (**C**) Immunohistochemical staining showed diffuse positivity for synaptophysin. (**D**) A diffuse loss of RB1 expression. (**E**) The Ki-67 labeling index exceeded 90%. (**F**) A mild membrane expression of SSTR2. EUS-FNA, endoscopic ultrasonography-guided fine-needle aspiration; EUS-FNB, endoscopic ultrasonography-guided fine-needle biopsy; H&E, hematoxylin–eosin stain; Pap, Papanicolaou stain; SSTR2, somatostatin receptor 2; SYP, synaptophysin

Pathological findings of endoscopic ultrasonography-guided fine-needle aspiration (EUS-FNA) identified small cell-type neuroendocrine carcinoma (NEC). Cytological specimens stained with Giemsa and Papanicolaou showed several cell clusters composed of small, round atypical cells with naked nuclei and necrotic cells (**Fig. 3A**). Histological specimens stained with hematoxylin–eosin showed numerous hypercellular clusters of small, round atypical cells with hyperchromatic naked nuclei (with scant cytoplasm), along with extensive areas of necrosis (**Fig. 3B**). Immunohistochemically, atypical cells demonstrated diffuse positivity for neuroendocrine markers, including chromogranin A, synaptophysin (**Fig. 3C**), and Insulinoma-Associated Protein 1 (INSM1), as well as a diffuse loss of RB1 (**Fig. 3D**). Additionally, the Ki-67 labeling index exceeded 90% (**Fig. 3E**). Membrane expression of somatostatin receptor 2 (SSTR2) was either mild or obscure (**Fig. 3F**). The pre-treatment diagnosis was unresectable, locally advanced panNEC, with the clinical stage defined as cT4N0M0 (Union for International Cancer Control [UICC] 8th edition)^6)^. She began a course of chemotherapy with carboplatin (420 mg) and etoposide (80 mg/m^2^).

**Fig. 4:**
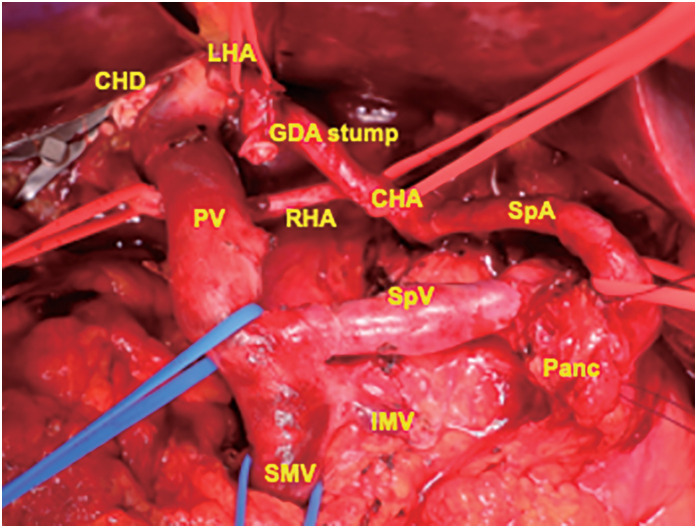
Operative findings after pancreatectomy. CHA, common hepatic artery; CHD, common hepatic duct; GDA, gastroduodenal artery; IMV, inferior mesenteric vein; LHA, left hepatic artery; Panc, pancreas; PV, portal vein; RHA, right hepatic artery; SMV, superior mesenteric vein; SpA, splenic artery; SpV, splenic vein

After 7 cycles of chemotherapy, an enhanced abdominal CT scan revealed a significant decrease in tumor size, which had reduced to 8 mm (**Fig. 1C**). The tumor was no longer in contact with the arteries, although some soft tissue density remained around the tumor (**Fig. 1D**). NSE had remained within normal limits since the second cycle of administration. The PET/CT showed decreased FDG uptake in the tumor, with a maximum standard uptake value of 3.0 (**Fig. 2B**). A pancreatoduodenectomy was planned as a conversion surgery. The major arteries, including CHA, SpA, and the gastroduodenal artery, could be separated at the layer where the adventitia was exposed, with no evidence of arterial invasion. Notably, compared with general cases of unresectable locally advanced pancreatic ductal adenocarcinoma (PDAC), arterial detachment in this case was relatively easier. Intraoperative rapid pathological diagnosis showed no evidence of malignancy in the peri-SpA plexus, peri-CHA nerve plexus, or periportal tissues, eliminating the need for a combined resection of vessels (**Fig. 4**). The operating time was 445 min, and the blood loss was 829 mL. Her postoperative course was almost uneventful, and she was discharged on postoperative day 16.

**Fig. 5:**
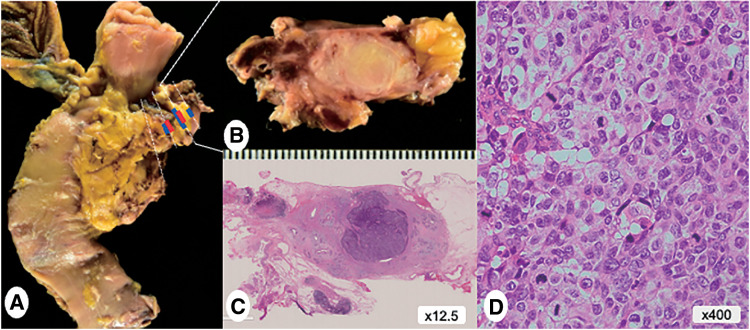
Pathological findings of the surgical specimen. (**A**) A residual tumor with a diameter of 8 mm at the pancreatic head–body junction. The red line indicates the NEC component, while the blue line represents the CIS component. (**B**) The tumor was relatively well-defined and localized, with the cut surface showing a yellowish-white solid mass. (**C**) The nodule showed a hypercellular tumor. (**D**) The tumor consists of large, round to polygonal cells with very slight necrosis. CIS, carcinoma *in situ*; NEC, neuroendocrine carcinoma

**Fig. 6:**
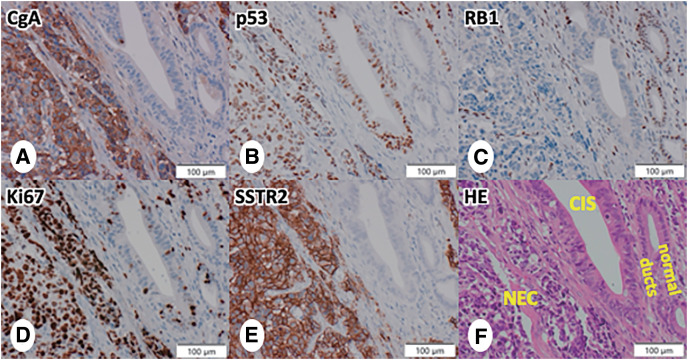
Immunohistochemical findings of the surgical specimen. (**A**) Diffuse positivity for chromogranin A. (**B**) Diffuse overexpression of p53. (**C**) Diffuse loss of RB1 expression. (**D**) The Ki-67 labeling index exceeded 90%. (**E**) Diffuse, prominent membrane expression of SSTR2. (**F**) A small amount of high-grade PanIN/CIS was observed around and inside the tumor, showing diffuse overexpression of p53 and diffuse loss of RB1. CgA, chromogranin A; CIS, carcinoma *in situ*; H&E, hematoxylin–eosin stain; NEC, neuroendocrine carcinoma; PanIN, pancreatic intraepithelial neoplasia; SSTR2, somatostatin receptor 2

**Fig. 7:**
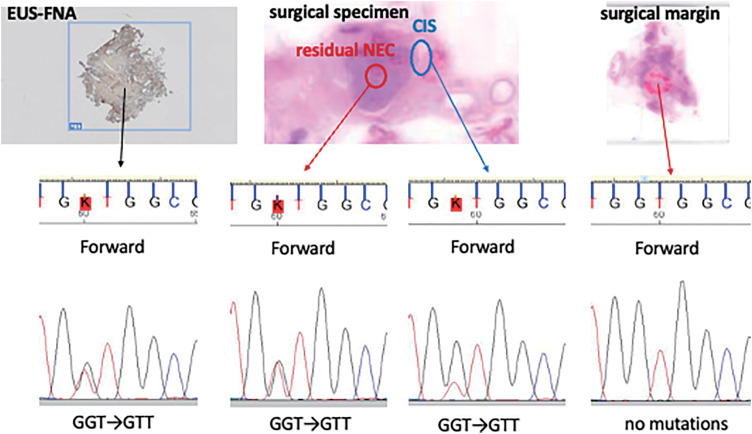
Sanger sequencing for detecting *KRAS* mutation in Exon 2. The same type of *KRAS* mutations (p. G12V) were detected in each component: small cell-type NEC from EUS-FNA, and large cell-type NEC and CIS from the resected specimen, but not in the surgical margin specimen. CIS, carcinoma *in situ*; EUS-FNA, endoscopic ultrasonography-guided fine-needle aspiration; NEC, neuroendocrine carcinoma

Pathological findings of the resected specimen revealed a residual tumor measuring 8 mm in diameter at the pancreatic head–body junction (**Fig. 5A** and **5B**). The tumor was relatively well-defined and localized, with a yellowish-white solid mass on the cut surface. Fibrosis and swollen lymph nodes were observed surrounding the tumor (**Fig. 5B**). Histologically, the nodule showed a hypercellular tumor (**Fig. 5C**) composed of large, round to polygonal cells with large nuclei (featuring vesicular chromatin) and abundant cytoplasm, forming nesting and solid structures (**Fig. 5D**). Mitoses were prominent, but necrosis was very minimal. In the fibrous stroma and swollen lymph nodes surrounding the tumor, fibrosis and granulation tissue, likely representing the site of tumor regression, were widely observed, with proliferation of fibroblasts, infiltration of small lymphocytes, infiltration of macrophages, and myxoid degeneration. Additionally, low-grade pancreatic intraepithelial neoplasias (PanINs) were scattered. Immunohistochemically, tumor cells were diffusely positive for pan-cytokeratin (antibodies AE1 and AE3) and several neuroendocrine markers (chromogranin A [**Fig. 6A**], synaptophysin, CD56, and INSM1). They were diffusely negative for Bcl10, GATA3, p40, and several hormones (insulin, glucagon, somatostatin, pancreatic polypeptide, gastrin, serotonin, and adrenocorticotropic hormone). Additionally, the tumor cells exhibited diffuse overexpression of p53 (**Fig. 6B**) and a diffuse loss of RB1 expression (**Fig. 6C**), and the Ki-67 labeling index exceeded 90% (**Fig. 6D**). Based on the histology and immunohistochemical findings, the tumor was diagnosed as large cell type NEC. Unexpectedly, tumor cells showed a diffuse, prominent membrane expression of SSTR2 (**Fig. 6E**). Furthermore, a small amount of high-grade PanIN/carcinoma *in situ* (CIS) was observed around and inside the tumor (**Figs. 5A** and **6F**), displaying a diffuse overexpression of p53 and a diffuse loss of RB1 (**Fig. 6B** and **6C**). The CIS extended to the cut end of the main pancreatic duct, but intraoperative examination of the true pancreatic margin specimen confirmed it to be negative (as noted later, no *KRAS* mutations were detected either) (**Fig. 7**). No extrapancreatic invasion or lymph node metastasis was identified (0/39), leading to a pathological stage of ypT1bN0M0 according to the UICC 8th edition staging.^6)^ Surgical margins were negative (R0), and the chemotherapy effect was evaluated as Score 2 (partial response) based on the modified Ryan scheme (College of American Pathologists [CAP] classification).^7)^

Genetic analysis using Luminex 100/200 technology (Luminex Corporation, Austin, TX, USA) on a formalin-fixed, paraffin-embedded tumor sample from the surgical specimen revealed a *KRAS* mutation (p.G12V). Furthermore, Sanger sequencing with microdissection confirmed that each component—small cell type NEC from EUS-FNA, as well as large cell type NEC and CIS from the resected specimen—shared the same *KRAS* mutation (p.G12V) (**Fig. 7**). This finding supported the final diagnosis of panNEC coexisting with CIS components, rather than indicating a collision nature.

She received adjuvant chemotherapy with 3 courses of carboplatin and etoposide, which was the same regimen as the preoperative treatment, and remained recurrence-free for 24 months after the pancreatectomy.

## DISCUSSION

This is a rare case of locally advanced, unresectable panNEC that was successfully treated with chemotherapy and subsequently underwent conversion surgery with a favorable outcome. The present case allowed us to report in detail the histogenesis and molecular behavior of panNEC and the changes in pathological findings of tumor cells before and after chemotherapy.

This case provides important histopathological and molecular insights. First, high-grade PanIN/CIS was observed around and inside the NEC component. In both components, a diffuse loss of RB1 and overexpression of p53 were observed. This suggests that the CIS components may have preceded or coexisted with the NEC components. Furthermore, the same type of *KRAS* mutation was detected in all lesions, including the small cell-type NEC from EUS-FNA and the large cell-type NEC and CIS from the resected specimen. These findings indicate that each lesion originated from the same pancreatic tissue, clearly supporting the current consensus that panNEC should be considered a subtype of PDAC, characterized by a high frequency of *KRAS* mutations.^8–10)^

Second, the predominant component of the tumor changed before and after chemotherapy. The EUS-FNA before chemotherapy showed predominantly small cell-type NEC with a tendency for extensive necrosis, whereas the residual tumor in the resected specimen after chemotherapy showed only a large cell-type NEC component with minimal necrosis. The contrast pattern of enhanced CT also shifted from a heterogeneously enhanced hypovascular tumor to a hypervascular tumor, reflecting the degree of necrosis. Although speculative, it is possible that the small cell-type NEC components, which originally constituted the majority of the tumor and were characterized by extensive necrosis, disappeared after chemotherapy, leaving only the large cell-type NEC component, which was initially mixed and resistant to chemotherapy. On the other hand, it is not yet clear whether the small cell-type NEC can convert to the large cell-type NEC due to chemotherapy or other factors.

Third, the diffuse membrane expression of SSTR2 was observed in the large cell-type NEC. Membrane expression of SSTR2 is usually seen in NETs, whereas it is absent or localized in NEC. Therefore, SSTR2 serves as an important marker for differential diagnosis and treatment selection between NETs and NEC.^11)^ In the present case, despite the presence of typical NEC, a diffuse and distinct membrane expression was observed, especially in the postoperative NEC component. It remains unclear whether this phenomenon is an exception or whether it occurs in a certain number of cases, and whether diagnosis and treatment (in combination with) targeting somatostatin receptors would be beneficial for NEC with SSTR2 membrane expression requires further case accumulation. Nonetheless, immunostaining for SSTR2 should be considered in NEC.

National Comprehensive Cancer Network (NCCN), European Neuroendocrine Tumor Society (ENETS), and North American Neuroendocrine Tumor Society (NANETS) guidelines^4,12,13)^ recommend platinum-based chemotherapy as adjuvant therapy for panNEC but do not recommend surgical resection or neoadjuvant therapy due to limited evidence. In the present case, platinum-based chemotherapy was administered as recommended for adjuvant therapy, without an intention of resection. There are very few case reports of surgical resection after chemotherapy for panNEC. **Table 1** shows the results of a search in PubMed (keywords: “pancreatic neuroendocrine carcinoma,” “pancreas,” “neoadjuvant treatment,” “pancreatectomy”).^14,15)^ All patients had small cell-type NEC and were treated with cisplatin and etoposide for 4–5 months as neoadjuvant therapy. The present case was unique in that the postoperative pathology was converted to large cell type NEC. If significant tumor reduction is achieved and radical resection is deemed feasible, surgical resection could be considered. Although panNEC rarely becomes resectable after chemotherapy, the patient had a favorable survival outcome following surgical resection, suggesting that a promising prognosis may be achievable when chemotherapy proves significantly effective.

**Table 1:** Reported cases of surgical resection after preoperative treatment for pancreatic neuroendocrine carcinoma

Author, year	Location	Preoperative pathological findings	Neoadjuvant therapy	Operation	Postoperative pathological findings	Adjuvant therapy	Survival
Li, et al. (2021)^14)^	Head and neck	Small cell type NEC Ki67 80%	EP (6c)	DP – CAR	Only necrotic and fibrotic tissues	None	8 m
Elzein, et al. (2021)^15)^	Head	Small cell type NEC Ki67 80%	EP (6c) → RT (50.4Gy)	PD + SMVr	NEC Ki67 5%	None	28 m
Our case	Head	Small cell type NEC Ki67 90%	CBDCCA + VP16 (7c)	PD	Large cell type NEC Ki67 80%	CBDCA + VP16 (3c)	21 m

CBDCA+VP16, carboplatin/etoposide; DP, distal pancreatectomy; DP-CAR, DP with celica axis resection; EP, etoposide/cisplatin; NEC, neuroendocrine carcinoma; PD, pancreatoduodenectomy; RT, radiotherapy; SMVr, superior mesenteric vein resection and reconstruction

## CONCLUSION

The present case represents a successful conversion surgery for locally advanced, unresectable panNEC after chemotherapy, revealing some interesting histopathological and molecular findings. Appropriate chemotherapy may enable surgical resection, and the effectiveness of this treatment could be reflected in postoperative survival outcomes. More relevant clinical cases need to be reported in the future to build evidence for the use of conversion surgery in panNEC.

## ACKNOWLEDGMENTS

We acknowledge all individuals who were involved in this study.

## DECLARATIONS

### Funding

No grant support or funding from public institutions or private enterprises was received for this case report.

### Authors’ contributions

YT drafted the manuscript.

MY supervised the writing of the manuscript.

HK and TS were involved in performing the surgery.

MY, HK, SO, YK, KO, RA, and TS contributed to patient management.

NO, TN, and TS made pathological diagnoses and performed molecular analyses.

All authors read and approved the final manuscript.

### Availability of data and materials

All data analyzed in this study are included in this manuscript.

### Ethical approval and consent to participate

All ethical and moral issues have been considered in this study. The present study was approved by the Institutional Review Board of Shizuoka Cancer Center (Approval Number: J2024-153-2024-1).

### Consent for publication

Written informed consent for the publication of this case report and the accompanying images was obtained from the patient.

### Competing interests

The authors declare that they have no competing interests.
